# Physicochemical and functional modifications induced by purple corn flour (*Zea mays* L.) and their sensory implications for loaf breads

**DOI:** 10.3389/fnut.2026.1850874

**Published:** 2026-06-12

**Authors:** Isabela da Mota Leal Lemos, Thaíza Rodrigues de Sousa, Carolina Pinto de Carvalho Martins, Luana Oeby de Oliveira, Carolyne Pimentel Rosado, Gisele Sanguia Sarandy, Jorge da Silva Pinho-Júnior, Marília Penteado Stephan, Anderson Junger Teodoro

**Affiliations:** 1 Nutrition Sciences Graduate Program (PPGCN), Faculty of Nutrition, Fluminense Federal University (UFF), Niterói, Brazil; 2 Integrated Food and Nutrition Center (CIAN), Faculty of Nutrition, Fluminense Federal University (UFF), Niterói, Brazil; 3 Nutrition and Dietetics Department, Faculty of Nutrition, Fluminense Federal University (UFF), Niterói, Brazil; 4 Food and Nutrition Graduate Program (PPGAN), Federal University of the State of Rio de Janeiro (UNIRIO), Rio de Janeiro, Brazil; 5 Food Biotechnology Laboratory, Department of Bromatology, Faculty of Pharmacy, Fluminense Federal University, Niterói, Brazil; 6 Brazilian Agricultural Research Corporation (EMBRAPA), Rio de Janeiro, Brazil

**Keywords:** consumer acceptance, food perception, functional foods, purple corn, sensory evaluation

## Abstract

**Introduction:**

The incorporation of functional ingredients, such as purple corn flour, into bakery products has attracted considerable attention due to its high content of bioactive compounds with potential health benefits. However, technological limitations and possible impacts on sensory acceptance still represent significant challenges. This study aimed to evaluate the effects of purple corn flour incorporation on the physicochemical, functional, and sensory properties of loaf bread, as well as to examine the relationship between compositional changes and consumer acceptance.

**Method:**

Five bread formulations were developed: one control formulation (100% wheat flour) and four formulations with partial replacement of wheat flour at levels of 15 and 30% using purple or yellow corn flour. The analyses included proximate composition, total phenolic content, antioxidant activity, and instrumental texture profile. Phenolic compounds were identified by high-performance liquid chromatography (HPLC). Sensory acceptance was evaluated by 100 consumers using a 9-point hedonic scale. Partial least squares regression (PLS-R) was applied to correlate compositional parameters with sensory responses.

**Results:**

The incorporation of purple corn flour, particularly at the 30% substitution level, significantly increased protein content (15.59 ± 1.33 g/100 g), dietary fiber (2.04 ± 0.05 g/100 g), total phenolic compounds (ranging from 58.22 ± 1.11 to 243.22 ± 9.57 μg GAE g^−1^), and antioxidant activity. HPLC analysis identified gallic and chlorogenic acids, with catechin as the predominant phenolic compound. Higher substitution levels resulted in increased hardness and adhesiveness. Sensory evaluation indicated that breads containing 15% substitution showed acceptance comparable to the control, whereas 30% substitution significantly reduced consumer preference. Multivariate analysis demonstrated that acceptance was positively associated with carbohydrate and energy content, while fiber, lipid, and moisture contents were negatively correlated with perceived quality.

**Conclusion:**

Moderate incorporation of purple corn flour represents a viable strategy to improve the nutritional and functional profile of bread while maintaining acceptable sensory quality. These findings reinforce the importance of balancing formulation strategies with consumer perception and support the application of purple corn flour in the development of functional bakery products.

## 1 Introduction

Bread is considered one of the oldest foods in human history and remains among the most widely consumed globally, primarily due to its broad accessibility and low production cost. Moreover, it represents a versatile food matrix with substantial potential for the incorporation of unconventional ingredients capable of providing additional health benefits to consumers (1).

Over the years, there has been a growing consumer demand for foods with health-promoting attributes, including the replacement of synthetic additives with naturally derived substances from plant sources (2). In response to this trend, the baking industry has increasingly invested in research focused on incorporating flours with functional properties into baked goods, aiming to enhance their nutritional value (3–5). As a result, several researchers have suggested the potential of enriching wheat flour with alternative crops, such as chickpea and colored wheat grains. In a study conducted by Gadalah and Aljebreen (6), the use of wheat flour blends supplemented with chickpea flour in bread production showed a positive correlation between increasing substitution levels and higher protein, crude fiber, and lipid contents, as well as greater dough resistance, indicating strengthening of the gluten network. This behavior may be associated with the presence of proteins and dietary fibers, which contribute to the structural stability of the dough. Furthermore, sensory evaluation demonstrated good acceptance of the formulations by consumers, with no undesirable characteristics identified when compared to the control bread. Koksel et al. (7) evaluated breads produced with whole colored wheat flours and observed that formulations prepared with blue and black wheat exhibited better crumb symmetry and cellular structure. Bread produced with blue wheat was distinguished by higher specific volume, lower firmness, and higher protein content, characteristics associated with improved technological performance in baking. In addition, the breads showed high mineral bioavailability, particularly for potassium, selenium, copper, and magnesium, highlighting their nutritional potential. Arslanhan and Karaoglan (8) developed gluten-free breads based on black chickpea flour supplemented with okra, fenugreek, and quince seed powders, ingredients selected for their functional properties and mucilage-forming capacity. The incorporation of these plant-based components improved the morphological structure of the breads and increased the dietary fiber content of the formulations.

Purple corn (*Zea mays* L.) is a grass of the Poaceae family, belonging to a group of flint corn varieties (*Z. mays* var. *indurata*; also referred to as Indian corn or calico corn), descending from a common ancestral variety known as “k’culli” in Quechua. Native to Peru and now widely distributed across markets in Asia, the United States, and Europe (9), purple corn is often classified as a “superfood” due to its remarkable polyphenol content and associated potential health benefits (10). In this regard, anthocyanins, primarily located in the cob and pericarp, are the most prominent phytochemicals in purple corn, mainly comprising cyanidin, peonidin-3-glucoside, and pelargonidin-3-glucoside derivatives (11). These compounds exhibit strong antioxidant capacity and function as protective agents against reactive oxygen species, which are implicated in degenerative processes linked to the onset of non-communicable chronic diseases such as obesity, diabetes mellitus, and cardiovascular disorders, often associated with oxidative stress (12).

Despite its promising health-related effects, the partial replacement of wheat flour with purple corn flour in loaf bread may result in undesirable sensory and technological characteristics, particularly regarding flavor and texture. In this context, the combined evaluation of instrumental and sensory data may provide a comprehensive approach for describing bread quality (13). These challenges must be addressed to develop products with suitable technological and instrumental properties and satisfactory consumer acceptance. Therefore, the aim of this study was to investigate how the incorporation of purple corn flour affects the sensory properties and consumer acceptance of loaf bread, and to relate these responses to changes in its technological and bioactive characteristics.

## 2 Materials and methods

### 2.1 Materials

Purple corn kernels were obtained through online purchase, while the remaining ingredients were acquired from local markets in Rio de Janeiro, Brazil. All materials were stored at the Integrated Center for Food and Nutrition (CIAN), Fluminense Federal University (Niterói, RJ, Brazil), under appropriate conditions until processing.

### 2.2 Bread production

**Figure 1:**
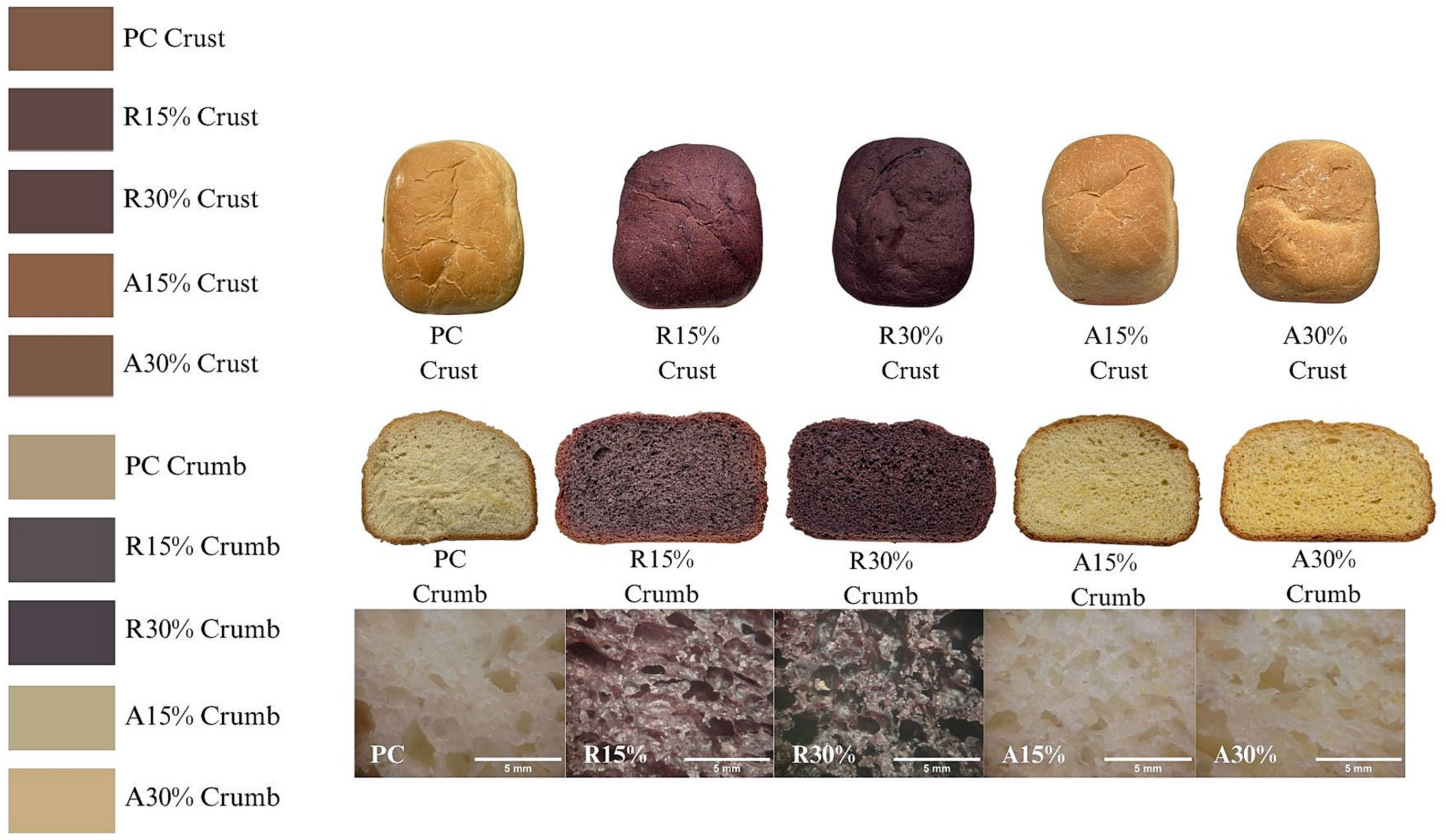
Colorimetric comparison of the color perceived through photographic records, the color measured by a colorimeter, and microscopy images of the crumb of the loaf bread formulations.

Purple corn kernels were washed, sanitized, and air-dried at room temperature, then milled (Pulverisette 14, Fritsch, Germany) without bran or germ removal. The flour was stored in light-free, hermetically sealed bags at −18 °C until use. Five loaf bread formulations were prepared: control (100% wheat flour, PC), 15 and 30% wheat flour substitution with purple corn flour (R15%, R30%, respectively), and 15 and 30% wheat flour substitution with yellow corn flour (A15%, A30%, respectively; Table 1). A domestic bread machine (Multipane, 127 V, 550 W, Britânia Eletrônicos S. A, Brazil) was used, adding ingredients in the order: water, olive oil, salt, wheat flour, corn flour, sugar, yeast. The selected cycle was the “basic bread” program, consisting of two mixing phases (10 min and 15 min) separated by a 20 min resting period, followed by an additional 30 s of mixing and 45 min of resting for fermentation and dough development, and subsequently baking for 1 h and 5 min, totaling 3 h. The baked breads (Figure 1) were stored at room temperature in plastic containers.

**Table 1:** Formulation of control, purple corn, and yellow corn breads.

Ingredients (g)	Samples				
	PC	R15%	R30%	A15%	A30%
Wheat flour	360	306	252	306	252
Corn flour	0	54	108	54	108
Water	200	200	200	200	200
Olive oil	25	25	25	25	25
Sugar	14	14	14	14	14
Yeast	6	6	6	6	6
Salt	7	7	7	7	7

PC, Control Bread; R15%, Bread with 15% purple corn flour; R30%, Bread with 30% purple corn flour; A15%, Bread with 15% yellow corn flour; A30%, Bread with 30% yellow corn flour.

### 2.3 Physicochemical characterization

#### 2.3.1 Centesimal composition

Moisture, ash, protein, lipid, and total dietary fiber contents were determined in triplicate according to the protocols of AOAC (14) and Instituto Adolfo Lutz (15) protocols, and all results were expressed as g/100 g on a dry weight basis (DW). Carbohydrate content was calculated by difference: 100 minus the sum of ash, protein, lipid, and fiber percentages. Water activity was measured using a portable water activity meter (LabStart-a_w_, Novasina AG®, Switzerland).

#### 2.3.2 Colorimetric evaluation

Color parameters were determined in triplicate using a digital colorimeter (Delta Vista Color®, model 450 G, Rio Grande do Sul, Brazil) according to the CIE Lab* system, measuring L* (lightness), a* (red/green), and b* (yellow/blue). The total color difference (ΔE) was calculated using Equation 1.


$$\mathrm{\Delta E}=\sqrt{(L^{*}-L_{0}^{*})+{\left(a^{*}-a_{0}^{*}\right)}^{2}+{\left(b^{*}-b_{0}^{*}\right)}^{2}}$$ (1)

### 2.4 Total phenolic content and antioxidant activity

Sample extracts (0.2 g/mL) were prepared in distilled water and 70% ethanol (v/v), vortexed (model NI 1059, Novainstruments, Brazil) for 3 min, sonicated (Cristófoli, 42 kHz, Paraná, Brazil) for 10 min, centrifuged (model 80-2B Centribio, Brasil) at 3,000 rpm, and the supernatant collected. Residues were re-extracted twice under the same conditions. Total phenolic content (TPC) was measured by the Folin–Ciocalteu method using gallic acid as standard, with results expressed as μg gallic acid equivalents/g sample (16). Antioxidant activity was determined by DPPH (1,1-difenil-2-picrilhidrazil) (17), Ferric Reducing Antioxidant Power (FRAP) (18) and ABTS•+ (19) using a spectrophotometer (SpectraMax i3x Multi-Mode Microplate Reader, Califórnia, EUA).

### 2.5 Phenolic compound identification by high-performance liquid chromatography

High-performance liquid chromatography (HPLC) analysis was performed on a Shimadzu® (Japan) system with LC-20AT pump, SPD-M20A diode array detector, CBM-20A control unit, DGU-20A5 degasser, and SIL-20 AC autosampler. Extracts, prepared as described using 80% (v/v) HPLC-grade methanol, were separated on a C18 reversed-phase column (Shim-pack VP-ODS, 5 μm, 250 × 4.6 mm) following INADA et al. (20). Phenolics were identified by comparing retention times and UV spectra with standards (caffeic, synaptic, ferulic, gallic, 4-hydroxybenzoic, p-coumaric, 5-O-caffeoylquinic acids; catechin; 3,4-dihydroxybenzoic acid; quercetin). Quantification used external calibration, with data processed in LC Solution (Shimadzu®, v1.25, 2009).

### 2.6 Instrumental texture profile analysis

Texture profile analysis of the crumb was performed using a TA. XT plus texture analyzer (Stable Micro Systems, Surrey, England) according to the method described by Scheuer et al. (21), with modifications. Crust hardness was evaluated following the methodology described by Bouaziz et al. (22), also with adaptations. For crumb TPA, 10 replicates were carried out using cylindrical samples (2.0 cm in height and 2.5 cm in diameter). The analyzer was equipped with a cylindrical aluminum probe (P/36R), with pre-test speed of 5 mm s^−1^, test speed of 2 mm s^−1^, post-test speed of 5 mm s^−1^, and a trigger force of 5 g. The textural parameters obtained were hardness, resilience, cohesiveness, gumminess, adhesiveness, springiness, and chewiness of the crumb. For crust hardness analysis, six replicates were performed directly on the crust using a 5 mm cylindrical probe (P/5R), with 7 mm penetration, a test speed of 1 mm s^−1^, pre-test and post-test speeds of 10 mm s^−1^, and a trigger force of 5 g.

### 2.7 Microscopic evaluation

Bread slices (1 cm thick) were observed under a stereomicroscope (Kimik) at 30x magnification. Images were captured using an Asus Zen Phone 9 camera in manual mode (ISO-100, f/1.9, shutter speed = 1/200 s). Scale bars were added using ImageJ software (version 1.50b, NIH), calibrated with a stage micrometer.

### 2.8 Sensory analysis

This study was approved by the Research Ethics Committee of the Faculty of Medicine, Fluminense Federal University (protocol number 69895223.5.0000.5243). Participants met the inclusion criteria (voluntary participation, age ≥18 years, and informed consent) and were excluded if they had dietary restrictions related to the ingredients used. A preliminary questionnaire was used to characterize the panelists in terms of gender, age, and consumption habits of corn-based bread. For the acceptance tests, each participant received samples coded with random three-digit numbers, presented monadically and in balanced order to avoid carry-over effects (23). A structured 9-point hedonic scale was used to evaluate appearance, aroma, flavor, texture, and overall impression, where 9 = “like extremely,” 5 = “neither like nor dislike,” and 1 = “dislike extremely” (24). Participants were informed that it was not mandatory to consume the entire sample, and mineral water at room temperature was provided between samples to cleanse the palate and neutralize residual flavors.

### 2.9 Statistical analysis

Data were expressed as mean ± standard deviation, based on triplicate experiments (n = 3). Statistical analyses were conducted using ANOVA, followed by Tukey’s test for mean comparisons, with a significance level of *p* < 0.05. Partial Least Squares Regression (PLS-R) was applied to explore the relationship between proximate composition parameters (independent variables) and acceptance scores (dependent variable). The analyses were performed using XLSTAT software (version 2022.1.2.1288; Addinsoft, Paris, France).

## 3 Results

### 3.1 Physicochemical characterization and colorimetric evaluation

The proximate composition of the analyzed bread samples is presented in Table 2. All formulations containing corn flours exhibited a significantly higher moisture content (*p* < 0.05) compared to the control sample (15.77 ± 1.44%). The formulation with 30% substitution of purple corn flour showed significantly higher ash (2.86 ± 0.05 g/100 g) and protein contents (15.59 ± 1.33 g/100 g) (*p* < 0.05) compared to the other samples. No significant differences (*p* > 0.05) were observed in lipid content among R30% (6.44 ± 0.64 g/100 g), A15% (5.63 ± 0.38 g/100 g), and A30% (6.24 ± 0.60 g/100 g), which presented the highest values. Fiber content was significantly higher (*p* < 0.05) in R30% (2.04 ± 0.05 g/100 g) and R15% (1.90 ± 0.06 g/100 g).

**Table 2:** Physicochemical composition of bread formulations.

Components (per 100 g DW)	PC	R15%	R30%	A15%	A30%
Moisture (%)	15.77 ± 1.44^a^	32.48 ± 1.38^b^	31.26 ± 0.67^b^	31.44 ± 0.79^b^	32.83 ± 0.65^b^
Ashes (g)	2.57 ± 0.03^b^	2.46 ± 0.06^ab^	2.86 ± 0.05^c^	2.30 ± 0.15^a^	2.30 ± 0.04^a^
Lipids (g)	5.40 ± 0.56^a^	5.46 ± 0.22^a^	6.44 ± 0.64^b^	5.63 ± 0.38^ab^	6.24 ± 0.60^ab^
Protein (g)	9.64 ± 1.69^c^	2.03 ± 0.11^a^	15.59 ± 1.33^d^	6.73 ± 0.25^b^	5.67 ± 0.15^b^
Fiber (g)	1.37 ± 0.17^ab^	1.90 ± 0.06^c^	2.04 ± 0.05^c^	1.60 ± 0.08^b^	1.30 ± 0.07^a^
Carbohydrate (g)	81.02 ± 1.76^b^	88.15 ± 0.25^d^	73.0 ± 1.44^a^	83.74 ± 0.55^bc^	84.57 ± 0.42^c^
Total energy (kcal)	411.26 ± 2.72^ab^	409.89 ± 0.75^a^	412.61 ± 2.54^ab^	412.53 ± 1.42^ab^	416.80 ± 2.43^b^
Water activity	0.82 ± 0.00^a^	0.85 ± 0.00^b^	0.91 ± 0.00^c^	0.91 ± 0.00^c^	0.91 ± 0.00^c^

All values are presented as means ± standard deviation (*n* = 3). Different lowercase letters within the same row indicate statistically significant differences (*p* < 0.05). Carbohydrate values were calculated by difference. DW, Dry Weight; PC, Control Bread; R15%, Bread with 15% purple corn flour; R30%, Bread with 30% purple corn flour; A15%, Bread with 15% yellow corn flour; A30%, Bread with 30% yellow corn flour.

Regarding carbohydrates, R30% (73.0 ± 1.44 g/100 g) showed the lowest values (*p* < 0.05), while R15% (88.15 ± 0.25 g/100 g) presented the highest (*p* < 0.05). In terms of total energy, A30% (416.80 ± 2.43 kcal/100 g) exhibited the highest values (*p* < 0.05), followed by PC (411.26 ± 2.72 kcal/100 g), R30% (412.61 ± 2.54 kcal/100 g), and A15% (412.53 ± 1.42 kcal/100 g), which did not differ statistically from each other (*p* > 0.05). Water activity (a_w_) was significantly lower (*p* < 0.05) in the control (0.82 ± 0.00) and R15% (0.85 ± 0.00), whereas R30%, A15%, and A30% showed similar values (0.91 ± 0.00) (*p* > 0.05).

Table 3 presents the colorimetric analysis of the crust and crumb of the analyzed bread samples. Regarding lightness (L*), values decreased (*p* < 0.05) with the addition of purple corn flour. The A30% crumb sample (72.45 ± 0.09) exhibited the highest lightness, indicating a lighter sample, whereas R30% and R15%, for both crumb (28.98 ± 0.09 and 34.50 ± 0.11, respectively) and crust (31.46 ± 0.07 and 33.17 ± 0.02, respectively), showed lower values, indicating darker samples. This was expected, given that purple corn flour is visually darker, as shown in Figure 1.

**Table 3:** Colorimetric analysis and classification of the total color difference of the crusts and crumbs of corn bread, using the control bread as a standard.

Sample	CRUMB				CRUST			
	L* (UA)	a* (UA)	b*(UA)	ΔE*	L*(UA)	a*(UA)	b*(UA)	ΔE*
PC	64,78 ± 0.05^c^	3,49 ± 0.17^c^	19,180 ± 0.06^c^	–	41,783 ± 0.03^d^	12,39 ± 0.07^d^	17,13 ± 0.04^c^	–
R15	34,50 ± 0.11^b^	4,46 ± 0.07^d^	-0,70 ± 0.06^b^	36.24	33,17 ± 0.02^b^	10,89 ± 0.08^b^	6,69 ± 0.09^b^	13.62
R30	28,98 ± 0.09^a^	5,42 ± 0.09^e^	-3,41 ± 0.03^a^	42.38	31,46 ± 0.07^a^	9,94 ± 0.10^a^	3,77 ± 0.06^a^	17.07
A15	69,61 ± 0.02^d^	0,46 ± 0.03^a^	19,51 ± 0.06^d^	5.71	45,09 ± 0.03^e^	14,96 ± 0.08^e^	21,61 ± 0.05^e^	6.13
A30	72,45 ± 0.09^e^	2,51 ± 0.02^b^	27,29 ± 0.16^e^	11.20	41,17 ± 0.04^c^	11,65 ± 0.07^c^	17,84 ± 0.06^d^	1.19

All values are expressed as means ± standard deviation (*n* = 3). For all parameters, samples differed significantly (*p* < 0.05). UA, unitless; PC, Control Bread; R15%, Bread with 15% purple corn flour; R30%, Bread with 30% purple corn flour; A15%, Bread with 15% yellow corn flour; A30%, Bread with 30% yellow corn flour.

However, higher values (*p* < 0.05) for the red color parameter (a*) were observed in the crust of the samples, with the highest values found in A15% (14.96 ± 0.08) and PC (12.39 ± 0.07). Conversely, lower values (*p* < 0.05) for the blue color parameter (b*) were found in the crumb of R30% (−3.41 ± 0.03) and R15% (−0.70 ± 0.06), indicating that a greater addition of purple corn flour is associated with a stronger tendency toward blue hues. In contrast, the samples A30% crumb (27.29 ± 0.16) and A15% crust (21.61 ± 0.05) showed higher values (*p* < 0.05), indicating a greater tendency toward yellow.

Regarding the perceived color difference, a very large difference was observed between the control and R15%, R30%, and A15% for the crust, as well as R15%, R30%, and A30% for the crumb.

### 3.2 Total phenolic content, antioxidant capacity, and HPLC phenolic profiling

Table 4 presents the results of total phenolic compounds (TPC) and antioxidant activity analyses for all extracts of the bread formulations. The ethanolic extract of sample R30% (243.22 ± 9.57 μg GAE g^−1^) showed the highest TPC (*p* < 0.05), followed by the ethanolic extract of R15% (107.74 ± 7.57 μg GAE g^−1^). Although the aqueous extracts of R30% (58.22 ± 1.11 μg GAE g^−1^) and R15% (48.77 ± 1.69 μg GAE g^−1^) did not differ significantly from each other (*p* > 0.05), both exhibited higher values compared to the other samples. The aqueous extract of A30% (15.58 ± 0.68 μg GAE g^−1^) showed the lowest levels (*p* < 0.05).

**Table 4:** Analysis of total phenolic compounds and antioxidant activity by FRAP, DPPH and ABTS methods of bread formulations.

Samples	Extractors	Antioxidant analisys			
		TPC (μg GAE g^−1^)	FRAP (μmol Fe_2_SO_4_/g)	DPPH (μmol TE/g)	ABTS (μmol TE/g)
PC	H_2_O	36.09 ± 2.29^c^	8.60 ± 0.40^c^	6.91 ± 0.21^bc^	0.65 ± 0.07^a^
PC	ETOH	19.63 ± 0.41^ab^	13.43 ± 0.55^d^	5.81 ± 0.06^a^	0.87 ± 0.01^ab^
R15%	H_2_O	48.77 ± 1.69^d^	13.56 ± 0.17^d^	18.10 ± 0.08^f^	2.64 ± 0.15^e^
R15%	ETOH	107.74 ± 7.57^e^	19.48 ± 0.85^e^	17.08 ± 0.11^e^	3.45 ± 0.02^f^
R30%	H_2_O	58.22 ± 1.11^d^	21.21 ± 0.28^f^	19.04 ± 0.58^g^	3.27 ± 0.15^f^
R30%	ETOH	243.22 ± 9.57^f^	50.67 ± 0.72^g^	17.27 ± 0.17^e^	3.47 ± 0.06^f^
A15%	H_2_O	18.94 ± 0.35^ab^	2.02 ± 0.04^a^	6.21 ± 0.17^ab^	1.05 ± 0.07^bc^
A15%	ETOH	28.03 ± 0.56^bc^	3.71 ± 0.59^b^	6.38 ± 0.18^ab^	1.22 ± 0.11^c^
A30%	H_2_O	15.58 ± 0.68^a^	1.46 ± 0.15^a^	8.27 ± 0.51^d^	1.57 ± 0.13^d^
A30%	ETOH	28.17 ± 1.29^bc^	3.73 ± 0.24^b^	7.55 ± 0.18^cd^	1.29 ± 0.07^cd^

All values are expressed as means ± standard deviation (*n* = 3). Different lowercase letters in the same column indicate statistically significant differences (*p* < 0.05). PC, Control Bread; R15%, Bread with 15% purple corn flour; R30%, Bread with 30% purple corn flour; A15%, Bread with 15% yellow corn flour; A30%, Bread with 30% yellow corn flour; TPC, total phenolic compounds; GAE, gallic acid equivalents; TE, Trolox equivalent.

The results indicate that formulations containing purple corn generally exhibited promising antioxidant activity. In the FRAP assay, the ethanolic extract of R30% (50.67 ± 0.72 μmol Fe₂SO₄/g) showed the highest ferric reducing capacity (*p* < 0.05), followed by the aqueous extract (21.21 ± 0.28 μmol Fe₂SO₄/g). No significant differences (*p* > 0.05) were observed between the aqueous extracts of A15% and A30%, a trend also observed in their ethanolic extracts.

In the DPPH assay, the aqueous extract of R30% (19.04 ± 0.58 μmol TE/g) exhibited the highest radical scavenging activity, followed by the ethanolic extracts of R30% (17.27 ± 0.17 μmol TE/g) and R15% (17.08 ± 0.11 μmol TE/g), which were statistically similar (*p* > 0.05). No statistical differences (*p* > 0.05) were observed between the aqueous and ethanolic extracts of A15%. Lower activities (*p* < 0.05) were found in the ethanolic extract of PC (5.81 ± 0.06 μmol TE/g).

In the ABTS assay, the ethanolic extract of R30% (3.47 ± 0.06 μmol TE/g), the aqueous extract of R30% (3.27 ± 0.15 μmol TE/g), and the ethanolic extract of R15% (3.45 ± 0.02 μmol TE/g) did not show statistically significant differences (*p* > 0.05). The control sample presented the lowest values (*p* < 0.05).

Thus, considering that the Folin–Ciocalteu assay is an indirect quantitative method for phenolics, the sample extracts were further analyzed by HPLC, following previously described phenolic compound standards. The results obtained are presented in Table 5.

**Table 5:** Bioactive compounds present in bread formulations analyzed by HPLC.

Phenolic compounds (μg/g)	PC	R15%	R30%	A15%	A30%
Caffeic acid	–	3.53 ± 0.04^c^	8.36 ± 0.10^d^	0.75 ± 0.02^a^	0.92 ± 0.02^b^
Sinapic + Ferulic Acid	–	6.85 ± 0.02^c^	12.61 ± 0.07^d^	1.41 ± 0.00^a^	2.84 ± 0.03^b^
Gallic acid	32.25 ± 0.08^a^	107.31 ± 0.26^e^	57.07 ± 1.02^d^	53.96 ± 0.14^c^	38.41 ± 0.12^b^
4-OH-benzoic acid	–	2.08 ± 0.01^b^	3.12 ± 0.02^c^	–	1.07 ± 0.05^a^
p-coumaric acid	–	3.86 ± 0.01^c^	5.38 ± 0.10^d^	0.70 ± 0.02^a^	0.98 ± 0.01^b^
5-CQA	–	1.79 ± 0.04^a^	4.68 ± 0.04^b^	–	–
Catechin	83.68 ± 0.56^c^	85.43 ± 0.18^d^	88.59 ± 0.65^e^	72.27 ± 0.56^b^	61.66 ± 0.31^a^
3,4-di-OH-benzoic acid	3.92 ± 1.02^a^	27.69 ± 0.15^c^	37.44 ± 0.04^d^	3.62 ± 0.20^a^	5.45 ± 0.03^b^
Quercetin	–	9.02 ± 0.14^b^	9.47 ± 0.08^bc^	9.80 ± 0.09^c^	7.39 ± 0.30^a^

All values are expressed as means ± standard deviation (*n* = 3). Different lowercase letters in the same column indicate statistically significant differences (*p* < 0.05). PC, Control Bread; R15%, Bread with 15% purple corn flour; R30%, Bread with 30% purple corn flour; A15%, Bread with 15% yellow corn flour; A30%, Bread with 30% yellow corn flour.

Among the phenolic standards analyzed, samples containing purple corn flour exhibited notable concentrations of these compounds, whereas the control sample contained only three types of phenolic compounds, namely gallic acid, catechin, and 3,4-di-OH-benzoic acid.

Regarding gallic acid, the R15% sample (107.31 ± 0.26 μg/g) showed the highest values (*p* < 0.05) compared with the other samples, including R30% (57.07 ± 1.02 μg/g). However, R30% (4.68 ± 0.04 μg/g) exhibited significantly higher levels (*p* < 0.05) of 5-O-caffeoylquinic acid. Among the polyphenols, catechin was the most abundant compound in all samples, including the control. All samples with corn flour substitution showed the presence of quercetin, with the highest concentrations observed in A15% (9.80 ± 0.09 μg/g) and R30% (9.47 ± 0.08 μg/g) (*p* > 0.05).

### 3.3 Texture analysis

The results of the texture profile analysis of the produced breads are presented in Table 6. Hardness was higher (*p* < 0.05) for R30% (10.45 N) and the control (9.96 N), with no statistical difference between them, whereas A30% (5.46 N) showed the lowest values (*p* < 0.05), representing the softest sample, although it did not differ significantly (*p* > 0.05) from R15% (6.18 N). Regarding resilience, no statistical differences were observed among the samples (*p* > 0.05).

**Table 6:** Results of the texture profile of breads.

Samples	Hardness (N)	Resilience	Cohesivness	Gumminess (N)	Adhesivness	Springiness	Chewiness (N)	Crush hardness (N)
PC	9.96 ± 0.93^a^	1.39 ± 1.88^a^	0.40 ± 0.051^b^	4.06 ± 0.71^a^	61.10 ± 7.07^a^	0.99 ± 0.00^a^	4.05 ± 0.71^a^	5.80 ± 0.34^bc^
R15%	6.18 ± 1.02^bc^	0.290 ± 0.03^a^	0.52 ± 0.050^a^	3.21 ± 0.36^a^	37.51 ± 8.71^bc^	0.99 ± 0.00^a^	3.20 ± 0.36^a^	3.92 ± 0.50^d^
R30%	10.45 ± 2.05^a^	0.21 ± 0.03^a^	0.40 ± 0.048^b^	4.12 ± 0.80^a^	65.51 ± 18.36^a^	0.99 ± 0.00^a^	4.11 ± 0.79^a^	6.51 ± 0.56^b^
A15%	8.40 ± 1.171^ab^	0.66 ± 1.12^a^	0.41 ± 0.045^b^	3.42 ± 0.32^a^	54.25 ± 9.88^ab^	0.99 ± 0.00^a^	3.42 ± 0.32^a^	8,13 ± 0.32^a^
A30%	5.46 ± 1.34^c^	0.45 ± 0.54^a^	0.42 ± 0.062^b^	2.24 ± 0.32^b^	32.64 ± 11.86^c^	0.99 ± 0.00^a^	2.24 ± 0.32^b^	5.40 ± 0.35^c^

All values are expressed as means ± standard deviation (*n* = 3). Different lowercase letters in the same column indicate statistically significant differences (*p* < 0.05). PC, Control Bread; R15%, Bread with 15% purple corn flour; R30%, Bread with 30% purple corn flour; A15%, Bread with 15% yellow corn flour; A30%, Bread with 30% yellow corn flour.

Cohesiveness was higher in R15% (0.52) (*p* < 0.05), indicating a firmer structure, while the other samples did not show significant differences (*p* > 0.05). For gumminess, PC (4.06 N), R30% (4.12 N), R15% (3.21 N), and A15% (3.42 N) did not differ significantly (*p* > 0.05), whereas A30% (2.24 N) showed the lowest value, suggesting a texture that is easier to chew.

Adhesiveness was higher in R30% (65.51 N), PC (61.10 N), and A15% (54.25 N) (*p* < 0.05), with no statistical differences among them (*p* > 0.05). In contrast, A30% (32.64 N) showed the lowest value, not differing from R15% (37.51 N) (*p* > 0.05).

Regarding chewiness, A30% (2.24 N) presented the lowest value (*p* < 0.05), indicating a softer texture and easier mastication, while the other samples did not differ significantly (*p* > 0.05), suggesting greater resistance to chewing.

Crust hardness measures the firmness of the outer layer of the bread, with A15% (8.13 N) being the sample with the hardest crust and R15% (3.92 N) the softest. No significant statistical differences (*p* > 0.05) were observed for resilience and Springiness.

Figure 1 shows the corn bread formulations in cross-section and observed by microscopy. The microstructural analysis revealed larger and more irregular alveoli in R30%, whereas R15% exhibited a more uniform crumb structure. As the proportion of purple corn flour in the formulation increased, an intensification of bread coloration, an increase in alveoli size, and a reduction in the final product volume were observed.

### 3.4 Sensory evaluation

In the sensory analysis, 74% of the 100 evaluators were female (mean age: 27.7 years; 63% aged between 18 and 25 years). Table 7 presents the results of the acceptance test using a hedonic scale. The mean scores of all formulations with added corn flour ranged from 6 (“liked slightly”) to 7 (“liked moderately”) for all attributes, while the control formulation remained at 7 (“liked moderately”).

**Table 7:** Sensory evaluation of breads assessing the parameters of color, aroma, texture, flavor, and overall acceptance.

Samples	Sensory analysis				
	Color	Aroma	Texture	Flavor	Global acceptance
PC	7.52 ± 1.40^a^	7.12 ± 1.53^a^	7.64 ± 1.54^a^	7.39 ± 1.44^ab^	7.55 ± 1.29^a^
R15%	6.72 ± 2.06^b^	7.02 ± 1.47^a^	7.01 ± 1.51^ab^	7.34 ± 1.37^ab^	7.37 ± 1.20^ab^
R30%	7.15 ± 1.92^ab^	6.86 ± 1.84^a^	6.18 ± 1.97^c^	6.85 ± 1.89^b^	6.86 ± 1.71^b^
A15%	7.54 ± 1.28^a^	7.07 ± 1.47^a^	7.04 ± 1.51^ab^	7.52 ± 1.23^a^	7.53 ± 1.12^a^
A30%	7.56 ± 1.32^a^	7.08 ± 1.49^a^	6.45 ± 1.77^bc^	6.91 ± 1.79^b^	6.97 ± 1.43^b^

All values are expressed as means ± standard deviation (*n* = 3). Different lowercase letters in the same column indicate statistically significant differences (*p* < 0.05). PC, Control Bread; R15%, Bread with 15% purple corn flour; R30%, Bread with 30% purple corn flour; A15%, Bread with 15% yellow corn flour; A30%, Bread with 30% yellow corn flour.

Regarding color, the samples A30% (7.56), A15% (7.54), PC (7.52), and R30% (7.15) were the most accepted, with no significant differences among them (*p* > 0.05). In terms of texture, the samples PC (7.64), R15% (7.01), and A15% (7.04) received the highest scores (*p* < 0.05) and did not differ significantly from each other (*p* > 0.05), whereas A30% (6.45) and R30% (6.18), which had higher levels of corn flour addition, were less accepted (*p* > 0.05).

For aroma, all samples were statistically similar (*p* > 0.05). Regarding flavor and overall impression, the highest scores were observed for PC, R15%, and A15%, with no statistical differences among them (*p* > 0.05). The samples R30% and A30% were the least accepted for these attributes (*p* > 0.05).

**Figure 2:**
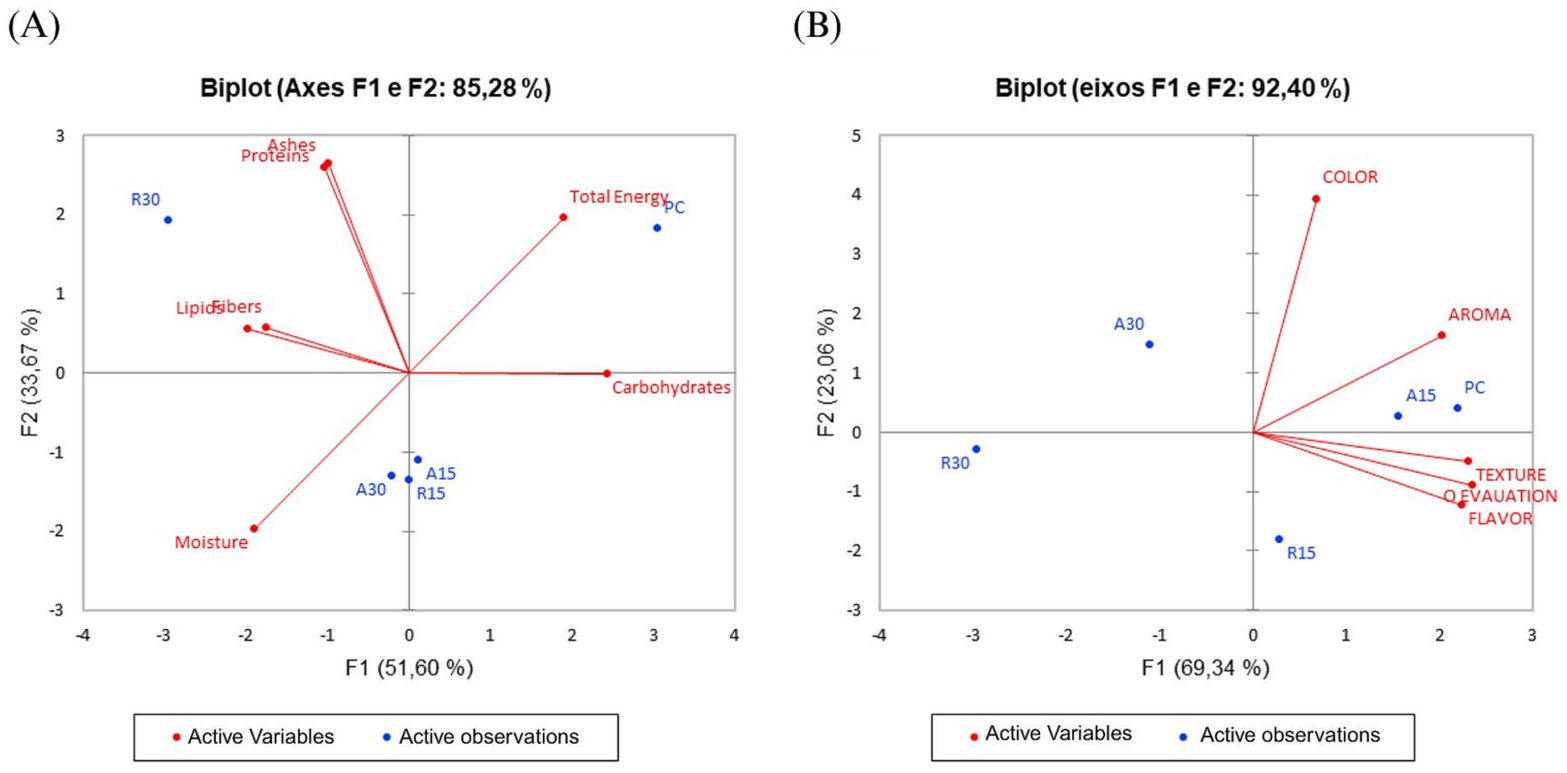
Biplots obtained by principal component analysis (PCA), based on physicochemical composition variables **(A)** and sensory acceptance attributes **(B)** of the sliced bread formulations.

Partial least squares regression (PLS-R), together with the biplots from Principal Component Analysis (PCA), enabled the exploration of the relationship between the physicochemical composition of the formulations and the sensory acceptance data. The first two principal components explained 85.28% of the total variance in compositional variables (Figure 2A) and 92.40% of the variance associated with sensory data (Figure 2B), indicating a high explanatory power of the generated models.

Among the compositional parameters, carbohydrate content stood out with a strong positive correlation with overall acceptance (*r* = 0.787) and very strong correlations with texture (*r* = 0.938) and aroma (*r* = 0.843). Consistently, caloric value also showed relevant positive correlations with texture (*r* = 0.721), reinforcing the influence of energy-dense constituents—especially carbohydrates—on the perception of sensory quality. In the biplot shown in Figure 2A, the control formulation (PC) was positively associated with carbohydrates and caloric value, corroborating its superior sensory performance.

On the other hand, moisture content showed a strong negative correlation with texture (*r* = −0.751) and consistently negative values for the other sensory attributes, indicating that formulations with higher moisture content were less accepted. This result may be associated with an excessively moist or poorly structured texture, compromising crumb quality perception.

Lipid content showed very strong negative correlations with flavor (*r* = −0.924), texture (*r* = −0.935), and overall acceptance (*r* = −0.954). These findings suggest that high lipid concentrations may negatively impact the sensory experience, possibly due to a greasy mouthfeel or interference with product structure and firmness. Similarly, fiber content showed significant negative correlations, particularly with aroma (*r* = −0.865) and color (*r* = −0.781), indicating potential adverse effects on sensory perception, possibly related to increased dough density and reduced crumb elasticity.

Based on Figure 2A, the R30 formulation, located in the upper-left quadrant, was associated with higher lipid and fiber contents, whereas the A30% formulation, located in the lower-left quadrant, was associated with higher moisture content—factors that may have contributed to their lower sensory acceptance.

Among the sensory attributes, flavor (*r* = 0.982) and texture (*r* = 0.933) showed very strong correlations with overall acceptance, highlighting their importance as key determinants of consumer preference. In contrast, color showed a weak correlation with overall acceptance (*r* = 0.105), suggesting that although it contributes to initial visual appeal, its impact on final product acceptance is limited.

Figure 2B, corresponding to the sensory acceptance analysis, shows a clear separation among formulations based on their performance across the evaluated attributes. The R15 sample, located close to the vectors corresponding to texture, flavor, and overall acceptance, stood out for its positive sensory performance.

## 4 Discussion

### 4.1 Effect of purple corn addition on bread composition and color

Regarding moisture content, Nascimento et al. (25) reported levels of approximately 34.52% in breads enriched with purple corn cob flour, values comparable to those observed in the corn-based bread formulations of the present study. High water activity favors nutrient availability for microorganisms, while also providing suitable conditions for gas exchange and an osmotically stable environment. These conditions facilitate microbial metabolism and waste release, promoting growth and contributing to product deterioration (26). Previous studies reported a_w_ values of 0.92–0.93 in whole-grain breads with jabuticaba peel (27) and 0.927–0.942 in breads containing carrot flour and jabuticaba-açu peel (28), which are consistent with the present findings.

A previous study on the proximate composition of *polvorón* made with blue corn flour reported a protein content of 8.04% (29). Another study analyzing Argentine purple corn flour found protein and ash contents of 10.2 ± 0.2% and 1.85 ± 0.03%, respectively (12). Both studies reported lower protein and ash values than those observed in the R30% formulation of the present study. Cabello-Olmo et al. (30), in their study on breads supplemented with a plant-based product composed of soy flour, alfalfa flour, barley sprouts, and viable microorganisms, reported that protein content reached 10.4, 11.3, and 12.1% as the level of the fermented product increased. A similar trend was observed in the present study, as R30% showed higher protein values compared to the control bread. Hosseini et al. (31) conducted a study to investigate the effect of corn germ flour and modified starch on the technological properties of Brotchen bread and reported lipid contents ranging from 1.2 ± 0.01% to 1.57 ± 0.02% and fiber contents from 2.52 ± 0.026% to 5.12 ± 0.11%, indicating lower lipid values but higher fiber contents when compared to the present study.

The incorporation of fiber-rich flours represents a technological challenge, as dietary fibers compete with wheat proteins for water, impairing gluten network formation. Nevertheless, the increasing demand for functional foods has driven the use of such ingredients due to their associated health benefits (32).

A study (33) that developed Ethiopian roasted maize bread made with wheat flour enriched with ginger reported energy content values ranging from 393.87 ± 1.46 to 436.17 ± 1.41 kcal, with the lowest value observed in the bread produced with 100% wheat flour, indicating results comparable to those of the present study.

Color is a critical parameter in baked products, as it is closely associated with sensory perception, aroma, and consumer preference. In addition to ingredients and additives, several physicochemical factors, including reducing sugar and amino acid levels, water content, pH, and processing conditions (such as time, temperature, and baking method) influence final color characteristics (34, 35). Furthermore, changes in sugar and protein contents, associated with caramelization and Maillard reactions during baking, may contribute to the development of darker coloration, as observed in formulations containing purple corn (36).

The color of corn kernels is associated with phenolic pigments—primarily anthocyanins in purple corn and carotenoids (lutein and zeaxanthin) in yellow corn—resulting in purplish/bluish hues in purple corn breads and yellow tones in yellow corn breads (10, 37–39).

### 4.2 Effect of purple corn addition on antioxidant capacity and phenolic profile of breads

Total phenolic content is widely considered a reliable indicator of *in vitro* antioxidant activity (36). Blanch and Ruiz del Castillo (40) reported a TPC of 48.1 mg GAE/100 g in homemade bread produced with 100% black corn flour, indicating higher values than those observed in the present study. However, it should be noted that the formulations evaluated herein contained only 15 and 30% purple corn flour.

Another study using 25% purple corn flour in bread formulation reported 178.1 ± 4.27 μmol TE/100 g in the DPPH assay, which is lower than the values obtained for both R15% and R30% in the present study (41).

It is important to highlight that phenolic compounds exhibit antioxidant activity primarily due to their radical scavenging and metal-chelating properties, in addition to presenting anti-inflammatory, antihypertensive, antidiabetic, antimutagenic, antibacterial, and antiviral effects (42). Thus, the higher levels of phenolic compounds and antioxidant activity observed in the formulations enriched with purple corn can be attributed to the high content of anthocyanins present in its composition—responsible for the characteristic purple pigmentation and strong antioxidant capacity (43) —in comparison with the control sample formulated exclusively with wheat flour. In addition to health benefits, antioxidant activity may also help delay bread staling (44).

In this context, Blanch and Ruiz del Castillo (40) also identified caffeic acid, quercetin, and chlorogenic acid in homemade black corn bread, supporting the present findings. Furthermore, Rodríguez et al. (12) analyzed phenolic compounds in Argentine purple corn flour by HPLC, reporting concentrations of 1.3 ± 0.1 μg/g for gallic acid and 6.0 ± 0.4 μg/g for ferulic acid—values lower than those observed in this study. Ma et al. (45) evaluated the use of a piston 3D printer and a screw 3D printer to produce black wheat steamed bread and reported values ranging from 3.89 ± 0.01 to 4.07 ± 0.00 μg·g^−1^ for quercetin and from 0.80 ± 0.76 to 3.07 ± 0.16 μg·g^−1^ for caffeic acid, indicating lower values than those found in R15% and R30%. In this regard, wheat-based bakery products, when enriched with natural bioactive compounds—such as those derived from purple corn—exhibit greater potential to promote health benefits, particularly due to their high antioxidant activity.

### 4.3 Effect of purple corn addition on bread texture

Instrumental texture profile analysis, which simulates mastication, is the most commonly used method for evaluating bakery products and is widely applied in the industry due to its correlation with potential sensory acceptance (46). Textural quality is associated with composition, structure, geometry, and physicochemical properties. Softer products generally exhibit lower firmness, hardness, gumminess (force required until swallowing), chewiness (time required to disintegrate until swallowing), and cohesiveness (internal resistance to rupture). In contrast, resilience (ability to recover shape after compression) and springiness (recovery of height after compression) should be maximized to improve textural quality (46, 47).

A study by Comettant-Rabanal et al. (41) on wheat breads enriched with whole purple corn flour reported hardness values ranging from 1.47 to 1.60 N with flour additions between 5 and 25%. In contrast, Tolve et al. (48), analyzing pigmented wheat breads enriched with grape pomace, observed significantly higher hardness values (21.8 to 23.2 N) compared to those found in the present study.

Do Nascimento et al. (32) evaluated breads produced with purple corn cob flour and hemicellulose, reporting cohesiveness and gumminess values of 62.81 and 2.70 N (5% corn flour), and 64.89 and 2.04 N (5% corn cob flour with 30 ppm hemicellulase), respectively. These values were higher than those observed in the present study, suggesting that the formulations evaluated herein exhibit lower resistance to disintegration (cohesiveness) and a less dense structure (gumminess).

Additionally, Do Nascimento et al. (32) reported chewiness values ranging from 2.45 to 1.93 N in breads containing purple corn flour and hemicellulose, indicating lower resistance to mastication compared to the present results.

According to Wang et al. (49), purple corn flour increases hardness, adhesiveness, and springiness while reducing elasticity and cohesiveness—a pattern also observed in this study.

From a microstructural perspective, the incorporation of purple corn flour affects gas retention and dough expansion during fermentation and baking, resulting in a crumb structure with irregular and non-uniform air cell distribution (49). This occurs because purple corn is gluten-free and may interfere with the formation of the protein network responsible for structure and elasticity (32, 50). Consequently, high levels of substitution may result in breads with lower specific volume, firmer texture, changes in flour and crust color, as well as modifications in flavor and sensory acceptance.

### 4.4 Effect of purple corn addition on sensory evaluation of breads

Canelo-Álvarez et al. (51) evaluated the acceptability of nixtamalized corn flour in whole wheat bread and reported scores of 4.00 for color and overall acceptability, and 3.50 for texture and flavor, indicating lower values compared to those observed in the present study.

A study assessing the sensory attributes of gluten-free muffins made with different types of corn (yellow, purple, and white) found that muffins prepared with yellow corn achieved high scores for appearance (8.2), flavor (8.2), and color (8.2), whereas those made with purple corn received lower scores for appearance (5.2), flavor (6.0), and color (5.4) (5). In this regard, the results for purple corn muffins were inferior to those obtained in the present study.

Another study evaluating the incorporation of 10% black carrot powder reported scores of 5.2 for texture, 6.3 for appearance, and 6.2 for overall acceptability. These lower values were attributed to a significantly darker color, which was perceived as less desirable by panelists (52).

In this context, the higher acceptance regarding texture for PC, R15%, and A15% may be associated with the preservation of dough structure at lower levels of wheat flour substitution, in which the gluten network likely remained sufficiently structured to ensure adequate softness, elasticity, and gas retention. In contrast, the A30% and R30% samples, which contained higher levels of substitution, showed lower acceptance, possibly due to the reduced formation of the gluten protein network, resulting in breads with higher density, firmer texture, and alterations in flour structure (13, 53).

Regarding aroma, the R30% and A30% formulations likely exhibited lower acceptance due to the intensification of residual flavors and modifications in texture (13, 32), factors that may negatively affect the overall consumer perception of the product.

Among compositional parameters, the strong association observed between carbohydrate content and sensory acceptance, particularly regarding texture and aroma, may be attributed to the role of carbohydrates in key technological properties such as structure formation, gas retention, and softness development, as well as their influence on sensory attributes like mild sweetness and aromatic profile. These findings are consistent with the literature, which identifies carbohydrates as important determinants of perceived quality in bakery products (54).

In the food industry, one of the main barriers to the acceptance of fiber-rich products is related to crumb color, flavor, and texture (55). The negative correlations observed between fiber content and aroma and color suggest that the high fiber content of purple corn flour may impair gluten network formation, consequently affecting texture and overall palatability (54).

The glycemic index (GI) of foods is primarily influenced by the rate of carbohydrate digestion and absorption, which may be affected by factors such as fiber content, starch structure, phenolic compounds, food matrix, and flour particle size (53, 56). Previous studies have demonstrated that the incorporation of pigmented cereals can modulate the GI of bakery products. Koksel et al. (7), evaluating breads produced with colored wheat grains, reported GI values ranging from 63.64 to 72.03, classifying blue wheat bread as medium GI and black, red, and purple wheat breads as high GI, although pigmented fractions reduced GI compared with commercial wheat bread. Similarly, Çetin-Babaoğlu et al. (57) observed lower GI values in snacks produced with blue corn, while Pruett (56) reported reduced GI values in muffins formulated with corn flour compared with sorghum flour formulations. In contrast, Jirarattanarangsri et al. (58) demonstrated that cooked purple waxy corn presented high GI values (95.8–97.2), likely associated with its elevated amylopectin content. Therefore, although pigmented cereals may contribute to nutritional and functional improvements, their effects on glycemic response remain dependent on formulation composition and processing conditions. In this context, a limitation of the present study is the absence of analyses related to glycemic index, bioaccessibility and bioavailability of anthocyanins and phenolic compounds, gastrointestinal digestive stability, shelf-life properties, and potential *in vivo* health effects of purple corn-enriched breads.

Overall, the findings of this study highlight the importance of integrating nutritional, technological, and sensory aspects in the development of bakery products enriched with purple corn. While the incorporation of this ingredient adds functional value, particularly in terms of bioactive compounds, higher substitution levels may negatively impact sensory and textural attributes. Therefore, these results provide relevant insights for optimizing formulations to achieve a balance between nutritional quality and consumer preference, supporting the development of more attractive and market-viable functional foods.

## 5 Conclusion

The incorporation of purple corn flour into loaf bread significantly modulated consumer sensory perception and acceptance, indicating that formulation level is a critical determinant of perceived product quality. Increased levels of incorporation resulted in higher phenolic content, antioxidant activity, and dietary fiber; however, these compositional modifications were associated with declines in sensory acceptance. In contrast, R15% yielded a more favorable balance between functional enhancement and sensory quality, achieving higher consumer acceptance without adverse effects on overall liking. No formulation was negatively evaluated. Multivariate analysis demonstrated that acceptance was positively associated with carbohydrate and energy-related attributes, whereas elevated lipid content, fiber, and moisture were negatively associated with perceived quality. Collectively, these findings highlight the importance of balancing nutritional enhancement and sensory quality in the development of functional bakery products. Future studies should investigate the bioaccessibility and bioavailability of anthocyanins and phenolic compounds, glycemic index, gastrointestinal stability, shelf-life properties, and potential *in vivo* health effects of purple corn-enriched breads to further support their functional and nutritional applicability.

## Data Availability

The raw data supporting the conclusions of this article will be made available by the authors, without undue reservation.
